# Deceleration Training in Team Sports: Another Potential ‘Vaccine’ for Sports-Related Injury?

**DOI:** 10.1007/s40279-021-01583-x

**Published:** 2021-10-29

**Authors:** Alistair J. McBurnie, Damian J. Harper, Paul A. Jones, Thomas Dos’Santos

**Affiliations:** 1 Department of Football Medicine and Science, Manchester United Football Club, AON Training Complex, Manchester, UK; 2 Department of Sport Science, School of Science and Technology, Nottingham Trent University, Nottingham, UK; 3 Institute of Coaching and Performance, School of Sport and Health Sciences, University of Central Lancashire, Preston, UK; 4 Human Performance Laboratory, Directorate of Sport, Exercise, and Physiotherapy, University of Salford, Salford, UK; 5 Department of Sport and Exercise Sciences, Musculoskeletal Science and Sports Medicine Research Centre, Manchester Metropolitan University, Manchester, UK

## Abstract

High-intensity horizontal decelerations occur frequently in team sports and are typically performed to facilitate a reduction in momentum preceding a change of direction manoeuvre or following a sprinting action. The mechanical underpinnings of horizontal deceleration are unique compared to other high-intensity locomotive patterns (e.g., acceleration, maximal sprinting speed), and are characterised by a ground reaction force profile of high impact peaks and loading rates. The high mechanical loading conditions observed when performing rapid horizontal decelerations can lead to tissue damage and neuromuscular fatigue, which may diminish co-ordinative proficiency and an individual’s ability to skilfully dissipate braking loads. Furthermore, repetitive long-term deceleration loading cycles if not managed appropriately may propagate damage accumulation and offer an explanation for chronic aetiological consequences of the ‘mechanical fatigue failure’ phenomenon. Training strategies should look to enhance an athlete’s ability to skilfully dissipate braking loads, develop mechanically robust musculoskeletal structures, and ensure frequent high-intensity horizontal deceleration exposure in order to accustom individuals to the potentially damaging effects of intense decelerations that athletes will frequently perform in competition. Given the apparent importance of horizontal decelerations, in this Current Opinion article we provide considerations for sport science and medicine practitioners around the assessment, training and monitoring of horizontal deceleration. We feel these considerations could lead to new developments in injury-mitigation and physical development strategies in team sports.

## Key Points

**Table:** 

High-intensity horizontal decelerations are performed frequently in team sport match play and possess unique biomechanical and physiological characteristics.
Team sport athletes need to be able to skilfully dissipate braking loads, develop mechanically robust musculoskeletal structures, and ensure frequent high-intensity horizontal deceleration exposure to accustom individuals to the potentially damaging nature of intense decelerations.
Horizontal decelerations are an important component of team sports athletic preparation, and more consideration is warranted for assessment, training and monitoring; it is hoped that the discussion presented in this article offers a sounding-board for future investigations and technological advancements that will be necessary to support the evolution of team sport.

## Introduction

Sprinting has been highlighted as a crucial component of conditioning practices in elite team sports, with its potential to ‘kill two birds with one stone’ in relation to both performance and injury mitigation [1–6]. Arguably, the main justification for this is that to prepare an athlete for the demands of competition, athletic training should seek to replicate or progressively overload these demands. No training method, however, stimulates the sprint-specific muscular activation patterns more readily than the action of sprinting itself [7, 8]. Despite this, the occurrence of near-to-maximal sprinting speeds (> 90% maximum sprinting speed; MSS) can be low during match play [9], and sprinting demands can vary between matches and positional roles [9–13]. Subsequently, supplementary sprint training is a highly recommended strategy in elite team sport environments to concurrently improve sprint performance and reduce injury risk [1, 2, 14]. This is vital to understand, as put simply, elite performers need to sprint to be prepared to perform in competitive match-play and have the resilience to cope with the game’s increasing high-intensity locomotor demands. This is why sprint conditioning is considered one of the key cornerstones of elite athletic training practices [1].

**Fig. 1:**
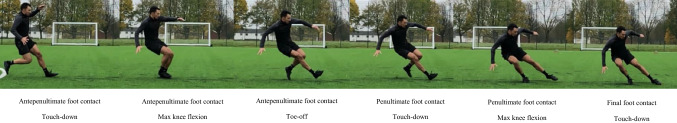
A photo sequence of the key spatiotemporal features during a pre-planned change of direction (COD) pivot manoeuvre. The steps preceding the final foot plant of COD play a key role in decelerating the system’s centre-of-mass for subsequent propulsion into the new intended direction. Not only does this facilitate effective COD performance, but it also serves to reduce multi-planar knee joint loading during the final foot plant. Further, during unanticipated CODs, the reduced time to make preparatory whole-body postural adjustments may potentially contribute to poor frontal and transverse kinetics and kinematics, and subsequently heighten the likely hazardous knee-joint loading experienced during the final foot plant

With this in mind, the authors would like to highlight another high-intensity locomotor action, which perhaps receives less attention: deceleration. Although being a generic term to describe a process performed in many athletic tasks, deceleration (i.e., negative acceleration) in the context of this discussion refers to the action performed during sporting scenarios that precedes a change-of-direction (COD) manoeuvre (Fig. 1) [15, 16] or an action immediately performed following a sprint to reduce momentum [17, 18]. Indeed, in most team sports (i.e., Australian Football, hockey, rugby league, rugby union, rugby sevens and soccer), high-intensity (< − 2.5 m/s^2^) decelerations appear to be performed more frequently than equivalently intense accelerations during competitive match play [17]. Crucially, high-intensity decelerations are linked to movement patterns commonly performed in match play, where athletes need to rapidly reduce momentum in order to evade or pursue opponents during offensive (e.g., ‘decelerate after a run in behind the opposition defence’) and defensive actions (e.g., ‘decelerate before performing a recovery run’) [19]. However, although a clear empirical link is yet to be established, high-intensity deceleration manoeuvres may need to be carefully monitored and managed, due to their propensity to generate high-impact braking ground reaction forces [20, 21], which may predispose lower-limb musculoskeletal structures to a heightened risk of neuromuscular and mechanical fatigue (i.e., accumulation of tissue damage, loss of stiffness and strength qualities) [18, 22]. Injuries can have long-lasting health, psychological and economic implications for both athlete and club (e.g., player salaries, cost of surgeries and rehabilitation) [18], and injury-risk mitigation strategies should be employed within organisations in order to maximise player availability, which is an important factor in sporting success [23, 24]. Accordingly, team sport athletes may need to be physically prepared and appropriately managed to cope with the high-intensity deceleration demands of competitive match play and training.

The physiological and biomechanical load-adaptation pathways appear to have distinct response rates, which may need to be characterised, trained and progressively overloaded differently within training cycles [25]. Therefore, although accelerations [26–28], decelerations [18, 28, 29] and high-speed running (HSR) [2, 3, 26, 27, 29–31] have each been highlighted as key performance indicators (KPIs) and expose individuals to high degrees of physiological and biomechanical stress, these stressors should be seen as fundamentally different [18, 25]. For example, accelerations may have a higher metabolic cost [32], whereas decelerations elicit higher mechanical demands (e.g., larger GRF peaks and loading rates), and thus, greater biomechanical loading [33]. This elevated mechanical load is likely explained by the often suddenly imposed nature of high-intensity decelerations, whereby rapid velocity reductions are enforced within constrained timeframes and spaces [34]. As a result, due to tactical evolutions of the modern game (i.e., increased high-intensity pressing, counter-pressing and counter-attacking) [35–37], team sport athletes may be exposed to an increased vulnerability of the muscle–tendon unit (MTU) properties in handling eccentric braking demands due to an increased necessity to perform more high-intensity accelerations and decelerations [35–37].

The purpose of this Current Opinion article is to highlight the potential importance of horizontal decelerations for both injury risk and performance in team sports. In addition, a physiological and biomechanical rationale for their inclusion as a KPI in a high-performance programme will be offered. We also provide sport science and medicine practitioners with considerations around the assessment, monitoring and training of horizontal decelerations. It is felt that these considerations could lead to new developments in injury-risk mitigation and physical preparation strategies in team sports.

## Biomechanical and Physiological Underpinnings of Deceleration

The performance of horizontal decelerations in team sports presents unique biomechanical (i.e., kinetic, kinematic and spatiotemporal) and physiological (i.e., metabolic, neural and MTU) characteristics. These attributes can vary depending on numerous factors, such as COD angle [16], approach velocity [16], match-play contextual factors [38], and type of external stimuli [39]. During COD, the preparatory deceleration steps require a posteriorly orientated braking impulse in order to rapidly reduce momentum prior to direction change [15, 16, 40, 41]. In this regard, particularly for sharper (angled) COD, the penultimate foot contact has been considered as a key ‘braking step’ for facilitating faster COD speed performance and alleviating potentially ‘high-risk’ knee joint loads [15, 16, 40–42]. Moreover, greater antepenultimate foot contact posterior and resultant braking forces were recently found to be associated with faster 505 COD performance, and are suggested to play an even more pivotal role in deceleration compared to the penultimate foot contact in the context of high-approach velocity 180° CODs, providing preparation time permits (i.e., time between stimuli and resulting action) [20]. This is particularly important, as players who ineffectively decelerate momentum prior to COD may experience increased knee joint mechanical loading during the final foot contact of COD [16]. When performing a sharp COD, the mechanical loading observed in horizontal deceleration is typically greater than what is observed during acceleration [43]. This is also the case when performing a maximal horizontal deceleration from a maximal sprint [34]. In both instances, the braking steps during deceleration have a unique ground reaction force profile characterised by a high impact peak force in a shorter time frame (i.e., ‘tall-thin’ force–time curve) [34]. This high-impact peak may be explained by postural differences seen between the heel strike associated with anterior foot placement during deceleration in contrast to a more mid- to fore-foot ground contact typically seen in acceleration [40, 41, 44–46]. Furthermore, a greater rate of change in velocity is typically experienced during deceleration tasks in which the high braking impulse required to reduce whole-body momentum must be applied in a shorter period of time (i.e., < 50 ms) [20, 34, 41]. As such, high angular velocities of the lower-limbs (e.g., ankle: 367 ± 192°/s, knee: 493 ± 252°/s [43]) are observed in the deceleration steps in which rapid triple flexion of the hip, knee and ankle joints are required for centre-of-mass (COM) lowering and effective orientation of horizontal braking forces [41].

A unique characteristic of performing horizontal decelerations is that to reduce momentum an emphasis on active muscle lengthening through braking action and the dissipation of mechanical energy is perhaps considered necessary [47]. The muscular contraction involved in these types of movements are commonly classified as ‘eccentric’ [48], and each of the contraction types (i.e., concentric, isometric and eccentric) involve different mechanisms of force generation at the contractile protein level [47]. In comparison to concentric and isometric muscle action types, eccentric muscle actions have the potential to generate greater forces for a given angular velocity [49]. Furthermore, eccentric muscle actions are more metabolically efficient, requiring less motor unit activation and oxygen consumption for a given muscle force [50]. In locomotion, it is a widely held assumption that this efficiency may be explained by the recycling of kinetic energy into elastic recoil energy from the tendons into the muscle fibres during limb support, resulting in less mechanical work and energy required during movement [51, 52]. However, during decelerations, the MTU may operate differently depending on the architecture of the muscle in question, which may influence the degree of eccentric muscle action. For example, particularly in distal muscle groups with long tendons (e.g., gastrocnemius-Achilles-soleus complex), these muscles may actually shorten to enable a compliant tendon to store, buffer and reduce the kinetic energy input to the muscle [53–57]. In contrast, in the more proximal musculature (e.g., quadriceps), the role of active lengthening and recycling of energy by the muscles may be greater due to reduced tendon lengths [54–58].

In resistance training, eccentric muscle contractions have been shown to be more effective in stimulating muscle strength gains [59], lead to regional hypertrophy [47], and induce positive shifts in muscle architecture [47]. Although still incompletely understood, it appears that the coupling of muscular fibre lengthening and heightened mechanical loading associated with eccentric muscle actions may create unique conditions underpinning the molecular mechanisms regulating the myogenic adaptations observed [47]. The mechanisms of structural remodelling appear to be contraction-specific, whereby eccentric-only resistance training results in markedly greater increases in fascicle lengths [60–62]. In contrast, greater changes are observed in pennation angle following concentric-only resistance training [60–62]. This likely reflects the differential addition of serial sarcomeres following eccentric resistance training, which has notable implications for performance and injury prevention, due to a concurrent shift in the ‘optimum’ force–length and force–velocity relationships of the working musculature, which subsequently alter their function [63–65]. In the context of high-velocity eccentric contractions, the evidence regarding physiological adaptation is limited. Following 10 weeks of ‘fast’ (e.g., 240°/s) or ‘slow’ (90°/s) isokinetic knee extension training, fascicle lengths of the vastus lateralis increased by 14% in the ‘fast’ training group compared to no significant changes in the ‘slow’ group [66]. Additionally, in animal models (i.e., rats), increased in-series sarcomeres have been observed after downhill running (i.e., heightened eccentric component) in contrast to uphill running (i.e., greater concentric component). Thus, it has been suggested that movement velocity, as well as contraction type, may be important regulators in the remodelling of contractile material placed in series [67]. This adaptation may be considered as a ‘protective’ mechanism after eccentric-induced muscle damage by limiting fascicle lengthening [67]. Potentially, this increases the maximum shortening velocities of muscle fibres [65] as well as the maximal forces produced at longer muscle lengths [63].

During horizontal deceleration a high degree of muscle pre-activation is required in order to effectively support the large external moments that are generated on ground contact [68]. Furthermore, during deceleration in the mid-eccentric phase of foot strike, peak levels of activation of the quadriceps have been reported to be far greater than those observed during maximal voluntary isometric contraction (161 ± 69%) [68]. Specifically, the high levels of activation may facilitate the production of high internal muscle forces over shortened time frames, subsequently allowing for the required external braking impulse to be generated to reduce horizontal momentum. Although also depending on muscle length and shortening velocity, the high peak levels of activation are necessary to produce the high internal knee extensor moments that are required to safely control and attenuate forces across knee joint flexion ranges executed across high angular velocities [68]. In addition, sub-optimal postures (e.g., trunk flexion), coupled with reduced hamstring activation during deceleration tasks (i.e., − 87 ± 84% peak quadriceps activation) may increase the risk of anterior displacement of the tibia, subsequently heightening the risk of anterior cruciate ligament (ACL) injury [68].

It has also been suggested that the unique properties of the tendons (i.e., series elastic component) within the MTU lead them to playing a key role in the dissipation of mechanical energy during deceleration actions (e.g., jump landings [54, 57], downhill running [69], enforced stopping [70]). It appears that the tendons may act as ‘mechanical buffers’ by reducing the rate of active muscle fascicle lengthening and peak force inputs (i.e., mechanical strain) to muscle fascicles [55]. Impact forces emanating from foot strike have been shown to increase during intense braking manoeuvres (i.e., rapid reduction of COM momentum within short stopping distances) [70] and from increasing jump heights [57]. Seemingly, regardless of this increase in intensity, the tendinous tissue still appears to withstand the majority of the MTU loading while the global fascicle lengthening remains unchanged [57]. However, as mentioned previously, this may be dependent on the muscle in question, with greater active lengthening demand appearing to occur in the vastus lateralis muscle than observed in the gastrocnemius medialis [57]. Thus, particularly in the more proximal musculature, the role of rapid pre-activation and isometric contraction of the muscles in preparation for ground contact also remains crucial, allowing for a higher gearing potential due to greater pennation angles during the force application [57]. Moreover, it has been shown that increased and rapid muscle activation during deceleration is critical for offsetting potential ligament loading of the knee during cutting tasks [71, 72]. As such, the combined functions of rapid pre-activation ability [55, 57, 71, 72] and mechanically robust tendons that act as ‘series-elastic shock absorbers’ [54] play critical roles in regulating the high eccentric forces experienced within the musculoskeletal system during high-intensity decelerations and protecting against injury [55].

## Implications for Injury Risk

Deceleration actions play a pivotal role when reducing whole-body momentum, particularly when running at high velocities and during the execution of sharp-angled directional changes [16]. However, such actions have been identified as mechanisms that are associated with non-contact ACL injury [73–75], due to their propensity to generate high multi-planar knee-joint loading (i.e., knee flexion, rotational and abduction moments) while the foot is planted [40, 76–79]. During match play, this may typically occur with externally directed attention (i.e., unanticipated COD manoeuvres) and opposition players in close proximity. These aspects may heighten potentially hazardous knee-joint loading, due to the increased task complexity and temporal constraints imposed during game scenarios [18]. In particular, because of the reduced time to make preparatory whole-body postural adjustments, the increased knee-joint loading during unplanned actions may be disproportionately greater than the muscle activation required to offset the adoption of potentially high-risk frontal and transverse plane kinematics (e.g., increased lateral trunk flexion and inadequate COM position) [80, 81].

Therefore, the multi-step nature of these actions places preliminary deceleration as a crucial strategy for reducing momentum and subsequent knee-joint loading during the final foot contact of COD (Fig. 1) and can be considered a modifiable risk factor for ACL injury mitigation. A key characteristic of this deceleration phase is the greater magnitudes of horizontal braking forces in the antepenultimate and penultimate foot contact relative to the final foot contact, coupled with greater knee flexion ranges of motion, which can reduce the subsequent knee joint mechanical loading experienced [15, 16, 40]. From a technical perspective, this is achieved by: (1) maintaining a low COM and anterior placement of foot to shift the base of support relative to the COM in order to increase posterior braking impulse, (2) ‘braking’ earlier and over multiple foot contacts to distribute loads, and (3) visual scanning and situational awareness to improve anticipation and increase preparatory times to facilitate the postural adjustments (points 1 and 2) and to moderate approach velocities [15]. Readers are directed to the following texts for a more detailed overview of developing optimal horizontal deceleration technique [15, 82].

Intense decelerations have the potential to induce muscle damage, as illustrated through the elevated levels of creatine kinase (CK; an indirect biomarker of muscle damage) during the 72-h period following repeated sprints with intense decelerations [83, 84]. Similar trends have been reported between the number of high-intensity deceleration actions and CK levels post-competitive match play in team sports, such as Australian Football (+ 129% increase on average) [28] and soccer [85, 86] players. In these instances, the eccentric braking force requirements of decelerations can impart damage on soft-tissue structures through high muscular tensions that can disrupt the structural integrity of the muscle fibres and result in myofibrillar degeneration, which may leak CK into the blood plasma [28]. Subsequently, over the course of a long competitive season, in which elite performers may be required to perform in match play every 3 days (e.g., fixture congestion), an under-prepared athlete may find themselves in a vicious cycle of ever-increasing neuromuscular fatigue and tissue damage, with the accumulation of tissue micro-trauma subsequently leading to chronically elevated CK levels [28, 87]. This will further diminish co-ordinative movement proficiency, leading to further tissue damage and increased injury risk that could negatively affect an individual’s capacity to skilfully dissipate braking loads [18]. With that said, it has been shown that the reduction of CK and associated detriments in neuromuscular performance may be possible when players have been accustomed to intense decelerations [88]. Crucially, the protective adaptations of a single bout of eccentric exercise can significantly dampen the damaging effects of subsequent eccentric bouts (i.e., the ‘repeated bout effect’) [89]. This can be primarily attributed to the development of sarcomeres in-series and improved distribution of strain across sarcomeres [67]. These components may be rapidly developed [27] and differ between trained and un-trained subjects [28, 29]. This may also be explained by the ‘fine-tuning’ of the neural activation patterns that enhance the protective effect of tendon elasticity [26]. Training interventions should, therefore, focus on these key aspects (Table 1) in order to effectively ‘vaccinate’ the athlete against the muscle damage-inducing effects of high eccentric loads that occur in competitive team sports [17, 18].

**Table 1:** Summary of the physiological and biomechanical properties of deceleration and the implications for team sport athletes from injury and performance perspectives

Theoretical rationale	Injury and performance considerations
Biomechanical	
↑ Magnitudes of horizontal braking impulse in the APFC and PFC relative to the FFC [15, 16, 40]	*Injury* ↓ Momentum and subsequent knee joint loading during steps prior to COD foot plant and subsequent ACL injury *Performance* ↑ Effective application of force for re-acceleration into new intended direction
↑ Impact peak forces and loading rates [34]	*Injury* ↑ Lower-body musculoskeletal loading *Performance* Rapid ↓ momentum in order to evade or pursue opponents based on impulse-momentum relationship
↑ Joint angular velocities of lower limbs [43]	*Injury and performance *↑ Eccentric power absorption and rapid limb positioning of all three lower-limb joints to facilitate braking over multiple foot contacts
Physiological	
↑ Forces for a given angular velocity during eccentric muscle actions compared to concentric or isometric [49–51]	*Injury* ↑ Forces lead to ↑ muscle damage and neuromuscular fatigue *Performance* ↑ Mechanical and metabolic efficiency for given work performed
↑ Quadriceps activation relative to MVC ↑ Hamstring activation levels relative to quadriceps (improving hamstring:quadriceps ratio) [68]	*Injury* ↑ Internal (muscle) moments to counteract large external joint moments during ground contact ↓ Risk of anterior displacement of the tibia through hamstring co-contraction, ↓ risk of ACL injury *Performance* ↑ Production of internal moments contribute to ↑ braking force (impulse)
↑ Pre-impact muscle activation ↑ Rate of eccentric force production [55, 71, 72]	*Injury* ↓ Rate of active muscle fascicle lengthening and eccentric/quasi-isometric force inputs (i.e., mechanical strain) to muscle fascicles *Performance* ↑ Ability to generate and attenuate rapid braking forces ↑ Technical ability to orientate braking forces
↑ Mechanical buffering capacity of tendon (stiffness qualities and precise neural activation patterns) [54, 55]	*Injury* ↓ Rate of active muscle fascicle lengthening and eccentric/quasi-isometric force inputs (i.e., mechanical strain) to muscle fascicles *Performance* ↑ MTU stiffness contributing to ↑ braking forces
↑ Co-coordinative proficiency and sensorimotor function [18, 99]	*Injury* ↓ Repetitive bouts of submaximal erroneous movement patterns and high-risk postures ↓ Likelihood of ‘mechanical fatigue failure’ *Performance* ↑ Movement skill and ability to perform sports-specific actions
↑ Positive architectural shifts in muscle fibres (i.e., ↑ sarcomeres in-series) and ↑ tissue tolerance to braking loads [88, 89]	*Injury* ↓ Muscle damage and negative effects of neuromuscular fatigue (i.e., repeated bout effect) *Performance* ↑ Expression of force–velocity characteristics

It is important to note that although horizontal decelerations may expose an athlete to an increased vulnerability through different mechanisms, it is frequent and optimised training that may offer a protective effect. As such, exposure to heightened mechanical loading is necessary to stimulate adaptation and protect the athlete against the damaging effects of high-intensity horizontal decelerations performed in competition

*APFC* antepenultimate foot contact, *PFC* penultimate foot contact, *FFC* final foot contact, *COD* change of direction, *ACL* anterior cruciate ligament, *MVC* maximal voluntary contraction, *MTU* muscle tendon unit

The long-term consequences of deceleration loads from an injury-risk perspective remain relatively unknown. However, research by Jaspers et al. [20] found that a higher cumulative loading of decelerations over 2–4 weeks led to an increased risk of overuse injury. A possible explanation for this relates to a ‘fatigue failure’ phenomenon discussed by Edwards [22], whereby the mechanical fatigue of biological tissue is propagated by damage accumulation as a result of repetitive loading and cumulative bouts of activity, which subsequently exceeds the remodelling rate of the tissue. Overuse injuries (e.g., tendinopathies and stress fractures) are defined by the concept of an injury occurring in the absence of a single, identifiable traumatic cause [90], and often manifest at points in the season of heightened training and game demand [31]. Here, the interactive effects of loading magnitude, which is dependent upon aspects such as activity type and intensity, and load cycles, may be more dependent upon activity duration, distance and repetition, which can collectively contribute to tissue degeneration over time [22].

Interestingly, in the study by Jaspers et al. [20], rapid 1-weekly increases in HSR (> 20 km/h) were related to an increased injury risk, findings that have been noted elsewhere [2, 3, 26, 27, 30, 31]. This perhaps sheds some light on the unique mechanisms of injury associated with deceleration loads. Deceleration-related injuries may manifest from a long-term aetiology in which a chronic imbalance between tissue degradation and remodelling may be present as a consequence of repetitive mechanical loads and an excess of eccentric muscle actions over longer periods of time in the absence of adequate recovery [28, 29]. Specifically, the delayed recovery timelines of the passive musculoskeletal structures (i.e., tendons, joint structures and bones), which have been shown to undergo heightened mechanical demands during deceleration actions [70], may be indicative of a more chronic overloading schema, and hence structural failure manifesting in the form of chronic injuries (e.g., stress fractures and tendinitis) [25]. In contrast, the acute ‘spikes’ in workload of HSR and their propensity to be associated with muscular-related injuries, such as hamstring strain injury [2, 91], is potentially indicative of a more short-term consequence. This may be driven in part by neuromuscular fatigue [92, 93], which has a more transient life cycle within the physiological system [94]. It is perhaps surprising, then, that fewer associations have been found between decelerations and injury in this regard [29]. It should be questioned whether the generalised nature of the currently available methods (i.e., whole-body accelerometery) [95] for monitoring deceleration loads are sensitive enough to determine such relationships [96], and a greater need for tissue-specific monitoring systems may be needed [97]. Indeed, discovering the optimal ‘Goldilocks’ load—not too much, but not too little—for tissue homeostasis will be key, in particular to support the management of the more chronic consequences of high-intensity decelerations, such as the ‘mechanical fatigue failure’ [22, 98] phenomenon.

## Considerations for the Integration of Horizontal Decelerations in Team Sports

It is in consideration of the above discussion that the authors would like to highlight the importance of another KPI within the weekly microcycle: high-intensity decelerations. Just as sprinting has rightly received much attention for its dual performance and injury-resilience benefits in team sports, the importance of high-intensity decelerations may need to be considered in the same light (Table 1). Consequently, horizontal deceleration may need to be seen as an important component in team sports high-performance environments that needs to be carefully assessed, trained and monitored accordingly. Thus, the following sections attempt to provide discussion on some of the more applied implications of horizontal decelerations in team sports, considering the theoretical rationale presented in the previous sections.

### Horizontal Deceleration Assessment

As mentioned previously, high-intensity decelerations are performed more frequently than equivalently intense accelerations across a number of team sports [17]. Yet considering the lack of available research on this topic, it appears the assessment of horizontal deceleration capabilities is much less prevalent in applied team sport settings. This may logically point towards the specificity and validity of sports-specific tests [100], which aim to replicate the manoeuvres commonly performed in the sport to inform training interventions and improve sports-specific fitness [101]. It is argued, however, that capacity-specific tests still add value to athletic assessment batteries for their ability to isolate underlying physical capabilities of athletes [101], and deceleration may be considered a key underpinning quality within a team sport athlete’s multidirectional speed profile [6, 15, 16].

With respect to horizontal deceleration ability, previous assessment methods have utilised high-speed cameras [102, 103], laser or radar technology [104–106], or satellite tracking systems [107]. By evaluating the performance of pre-planned decelerations over a range of distances (e.g., 5, 10, 15, and 20 m), a deceleration ‘gradient’ may be created and athletes may be identified who show poor performance in key metrics (e.g., peak/average deceleration and time/distance to stop) [104, 106]. Hypothetically, in this regard, an athlete performing a multi-component testing battery may demonstrate a reduced deceleration capacity while possessing advanced acceleration or maximum velocity capabilities. Therefore, it may be assumed that these identified athletes are likely to be underprepared for the physical demands of competitive team-sports, due to the even greater loading demands of performing unplanned decelerations in match play, particularly if they are a ‘faster’/‘concentric-dominant’ athlete who may approach deceleration tasks from increased movement speeds. With this information, coupled with the assessment of an athlete’s movement competency [102, 103] and physical capacity (particularly eccentric and reactive strength [108, 109]), practitioners may more readily characterise specific biomechanical or neuromuscular deficits within an athlete’s multidirectional speed profile and develop individualised training accordingly [6, 110].

### Horizontal Deceleration Training

Recent findings have shown promising results with respect to improving some of the technical and mechanical characteristics underpinning horizontal deceleration actions following targeted training. For example, two separate field-based training interventions [102, 111], comprising 2-weekly multidirectional speed sessions across 6 weeks, were both shown to be effective in reducing surrogates of ACL injury risk, as well as improving COD performance in team sport athletes. Specifically, this was achieved by either reducing high-risk technique deficits (i.e., factors associated with increased multi-planar knee-joint loading: lateral trunk flexion, extended knee postures, knee valgus, hip internal rotation and improved braking strategies) [102] or enhancing kinetic and kinematic characteristics associated with effective braking performance (i.e., horizontal propulsive forces, horizontal to vertical mean braking and propulsive force ratios for the penultimate and final foot contacts, final foot contact peak knee flexion and penultimate hip flexion angles) [111]. Importantly, horizontal deceleration training formed a fundamental training component in the early training weeks of both interventions, in which drills that provided ‘windows’ to decelerate were provided and key postures conducive to deceleration performance were encouraged throughout [102, 111]. In the later weeks, this focus was then divided into ‘shallow’ (i.e., < 90° COD) and ‘sharp’ (i.e., > 90° COD) emphases, where on the latter training day, deceleration was embedded into all large-angled cuts and pivots as a means of reinforcing key postures promoted in the preliminary sessions [102, 112].

In contrast, both the acute and the chronic physiological adaptations following field-based horizontal deceleration training is certainly an area that warrants future investigation. For example, recent work has shown improved sprint performance times and mechanics, as well as positive architectural shifts in the hamstring muscles (e.g., increased fascicle length), following high-velocity sprint training [113]. In this regard, questions exist as to whether similar outcomes would be observed following targeted horizontal deceleration training interventions. For example, due to the high eccentric braking demands of the anterior chain during braking, coupled with the role of the passive structures within the MTU, it may be expected that positive structural and neuromuscular adaptations can be induced. Furthermore, recent advancements have highlighted the deterioration of isokinetic eccentric hamstring strength following a 3-week period of detraining, whereas linear (10 m) and COD speed (10 m approach, 180° pivot and 10 m exit) performance times were maintained [114]. This highlights a potential shift in the force–velocity characteristics underpinning these movements following a period of training cessation, which may be of concern if compensatory strategies are adopted [115]. Thus, as well as revealing a better understanding into the minimum effective dosages of horizontal deceleration training, whether these adaptations provide a ‘protective’ effect (as potentially indicated by reduced markers of fatigue and muscle damage such as decreasing delayed onset of muscle soreness and circulating CK [88]) against injury over the long term in team sports is currently unknown and is another recommended avenue for further research.

### Horizontal Deceleration Monitoring

It is now common practice for team sports performance and medicine departments to closely monitor key variables through the use of tracking technologies (e.g., global positioning systems) in order to maintain players’ fitness qualities during athletic training exercises when appropriately identified [5, 14]. Further to this point, perhaps one of the key aspects to monitor with the advent of this technology is the volume of sprinting distance (e.g., running distances covering > 25.2 km/h) and the intensity of sprinting achieved in a week [2]. Empirical findings support this, indicating that athletes who accumulate large chronic training exposures to sprinting [2, 3], as well as regularly sprinting at near maximal intensities (i.e., > 95% maximum velocity) during training [2], have demonstrated a reduced risk of lower-limb injuries in comparison to their teammates who produced lower running intensities (i.e., < 85% maximum velocity) and lower sprint training volumes [2].

In the same regard, further research is required to clarify the specific monitoring and dosage requirements of very high-intensity decelerations (i.e., near maximal). The authors also criticise the application of the same arbitrary thresholds to classify ‘high-intensity’ mechanical activity (e.g., typically acceleration: > 3 m/s^2^; deceleration: < − 3 m/s^2^ [17]). The biomechanical and physiological differences of these measures have been discussed extensively in this paper [17, 18, 25, 34]; in addition, higher maximal deceleration values have been reported in comparison to acceleration values across all playing positions in soccer match play (e.g., 5.7–6.3 m/s^2^ vs. 4.4–4.7 m/s^2^) [116]. Thus, it is suggested that a more individualised approach is taken in which an individual’s maximal horizontal acceleration-deceleration profile is characterised, from which subsequent thresholds may then be applied and used to monitor deceleration loads in match play and training [37].

Nevertheless, advances in tracking technologies, coupled with the advent of machine learning techniques, could enable identification of structure-specific (e.g., segmental accelerometry [21] or markerless motion capture [117]) loads. Accordingly, these techniques may more precisely identify loading at the tissue level, differentiate between high-intensity actions, identify asymmetrical loading patterns between limbs, and pinpoint biomechanical and spatiotemporal parameters indicative of compensatory movement strategies performed, especially under fatigue. This kind of intelligence may be key to uncovering which short- and long-term factors are associated with an increased ‘injury risk’ when performing high-intensity locomotor actions like deceleration. Therefore, future investigations should aim to evaluate deceleration and its association with fatigue and injury risk with these questions in mind to better understand the mechanisms underpinning fatigue and injury, and, thus, identify key benchmarks to assess and monitor its development in athletes.

To conclude, future work in the area of horizontal deceleration is certainly warranted and it is hoped that these discussion points provide a sounding-board for future investigations and technological advancements that will be necessary to support the evolution of team sport, in particular with respect to the development and maintenance of players’ high-intensity locomotor profiles in the modern game [37]. Below, some summary recommendations are provided for future developments to improve the management of horizontal decelerations practices in team sport settings:

1. The evaluation in the field of deceleration capacity and technique should be included in athlete testing batteries in the same way that linear speed testing and jump testing are widely employed. This will enable the identification of individual maximal deceleration capabilities that will also permit individualisation of the deceleration loads that players have been exposed to.
2. The progressive development of an athlete’s horizontal deceleration ability, along with deceleration-focused resistance training, may need to be placed as a key cornerstone in any multidirectional speed-development programme. This should be delivered through an informed understanding of the mechanics necessary for successful performance and reducing relative injury risk.
3. The acute and chronic exposure of high-intensity decelerations should be monitored and periodised with an understanding of the unique physiological and biomechanical characteristics that separate them from other KPIs, such as acceleration and HSR. In particular, the measurement of structure-specific loading (i.e., muscles, tendons, joints and bones), which are known to have different recovery timelines, should be considered in the development of advanced monitoring systems.

## Conclusion

In summary, while the continued development and maintenance of high-velocity locomotor activity remains a vital piece of the ‘performance puzzle’ in team sports [1–6], it is advised that practitioners ‘do not speed up what an athlete cannot slow down’ [18]. Sports science and medicine practitioners should begin to monitor their athletes’ deceleration activity with more vigilance and appropriately progress training loads with this key feature in mind. During field-based activity, athletes may need to be regularly exposed to high-intensity deceleration within their weekly microcycle, and compensatory work could be recommended in the same way that HSR supplementation is now commonly utilised [5, 14]. Furthermore, horizontal deceleration should be trained as a movement skill in conjunction with improving an athlete’s eccentric strength capacity and neuromuscular performance qualities in off-field training.

It is hoped that the discussion covered in this article highlights the need to ‘mechanically protect’ team sport athletes from the damaging nature of high-intensity decelerations performed in their respective sports [18]. This should hold special relevance for those tasked with the challenging problem of pushing the boundaries of athletic performance while reducing injury risk.

## Declarations

### Author informations

Alistair J. McBurnie is a Sports Science Analyst at Manchester United FC, Manchester, United Kingdom. Damian J. Harper is a lecturer in Sports Coaching and Performance at the University of Central Lancashire, Preston, United Kingdom. Paul A. Jones is a lecturer in Sports Biomechanics and Strength and Conditioning at the University of Salford, Manchester, United Kingdom. Thomas Dos’Santos is a lecturer in Sport and Exercise Science at the Manchester Metropolitan University, Manchester, United Kingdom.
